# Complete Genome Sequence of Bacillus pumilus Strain WP8, an Efficient Plant Growth-Promoting Rhizobacterium

**DOI:** 10.1128/genomeA.01452-14

**Published:** 2015-01-22

**Authors:** Yijun Kang, Min Shen, Huanli Wang, Qingxin Zhao

**Affiliations:** a School of Life Science and Technology, Yancheng Teachers University, Yancheng, People’s Republic of China; b Jiangsu Key Laboratory for Bioresources of Saline Soils, Yancheng, People’s Republic of China

## Abstract

Bacillus pumilus strain WP8 is an efficient plant growth-promoting rhizobacterium. Here, we present the complete genome of WP8 and its genes involved in plant growth promotion and biocontrol.

## GENOME ANNOUNCEMENT

Bacillus pumilus WP8, a plant growth-promoting rhizobacterium (PGPR), was isolated from the rhizosphere of wheat (Triticum aestivum L.) in Yangzhou City, China (1). Both the growth-promoting and biocontrol potentials of B. pumilus WP8 have been proven to be efficient and widely adapted to multiple plant species and environments (2–4). Some potential mechanisms of action of B. pumilus WP8, such as the attenuation of virulence in pathogenic bacteria, still need to be explored further.

After DNA shearing with a Covaris M220, clone library construction, and bridge amplification, the whole-genome sequencing of WP8 was performed using Illumina MiSeq and Pacific Biosciences platforms. A total of 0.95 Gb of sequence was generated from a 300-bp paired-end library after optimizing the raw sequence data, giving 260.65-fold coverage of the genome. Meanwhile, 0.72 Gb of sequence was obtained from a PacBio 8- to 10-kb library. The filtered reads generated by Illumina sequencing were assembled by SOAP*denovo* version 2.04 (5), with which the sequence data from PacBio sequencing were adjusted. Gap filling between contigs and the remaining gaps between scaffolds were closed by using GapCloser version 1.12 and sequencing the PCR products. Annotation was performed using Glimmer 3.02 (6, 7), tRNAscan-SE version 1.3.1 (8), and Barrnap 0.4.2 (9). Putative proteins were searched against the Clusters of Orthologous Groups (COG) (10), nonredundant protein sequences (nr), string (11), and Gene Ontology (GO) (12) databases.

The complete genome sequence of WP8 is a circular 3,708,888-base chromosome with a G+C content of 41.57%. The number of putative genes totals 3,678, with an average G+C content 42.10% in the coding regions. Furthermore, 22 rRNA operons and 81 tRNAs were predicted. There are some genes associated with growth promotion and biocontrol, such as genes encoding capsular polysaccharides (QR42_15530) and sporulation transcription factors (QR42_00345) that are related to biofilm formation (13, 14), as well as genes encoding chemotaxis-related proteins (QR42_10155, QR42_15985, etc.) and cell wall degradation-related proteins (QR42_09230) that may be responsible for colonization. Relatively few genes are involved in antimicrobial biosynthesis in the genome besides a siderophore-synthetic gene (QR42_05295). Genes encoding endoglucanase (QR42_07950), protease (QR42_17905), and β-1,3-glucanase (QR42_02755) are considered to play important roles in the biocontrol of some fungal pathogens and nematodes (15–18). Genes encoding nonribosomal peptide synthetase (QR42_01955-QR42_01980), putative pumilacidin, may be responsible for the attenuation of virulence in some pathogenic bacteria and need to be studied. In addition, genes encoding arsenical pump membrane protein (QR42_01870), monooxygenase (QR42_08825), UV damage repair endonuclease (QR42_16900), catalase (QR42_18570), and stress proteins (QR42_01610, QR42_02315, etc.) enable WP8 to adapt to a wide range of environments (19–21).

### Nucleotide sequence accession number.

The complete nucleotide genome sequence of B. pumilus WP8 has been deposited in GenBank with the accession number CP010075.

## References

[1] Kang Y, Cheng J, Mei L, Yin S 2010 Screening and identification of plant growth-promoting rhizobacteria. Wei Sheng Wu Xue Bao 50:853–861. (In Chinese.). 20815230

[2] Kang Y, Shen M, Wang H, Zhao Q 2013 A possible mechanism of action of plant growth-promoting rhizobacteria (PGPR) strain *Bacillus pumilus* WP8 via regulation of soil bacterial community structure. J Gen Appl Microbiol 59:267–277. doi:10.2323/jgam.59.267. 24005176

[3] Kang Y, Shen M, Yang X, Cheng D, Zhao Q 2014 A plant growth-promoting rhizobacteria (PGPR) mixture does not display synergistic effects, likely by biofilm but not growth inhibition. Microbiology 83:666–673. doi:10.1134/S0026261714050166.

[4] Shen M, Jun Kang Y, Li Wang H, Sheng Zhang X, Xin Zhao Q 2012 Effect of plant growth-promoting rhizobacteria (PGPRs) on plant growth, yield, and quality of tomato (*Lycopersicon esculentum* Mill.) under simulated seawater irrigation. J Gen Appl Microbiol 58:253–262. doi:10.2323/jgam.58.253. 22990485

[5] Luo R, Liu B, Xie Y, Li Z, Huang W, Yuan J, He G, Chen Y, Pan Q, Liu Y, Tang J, Wu G, Zhang H, Shi Y, Liu Y, Yu C, Wang B, Lu Y, Han C, Cheung DW, Yiu SM, Peng S, Xiaoqian Z, Liu G, Liao X, Li Y, Yang H, Wang J, Lam TW, Wang J 2012 SOAPdenovo2: an empirically improved memory-efficient short-read *de novo* assembler. GigaScience 1:18. doi:10.1186/2047-217X-1-18. 23587118 PMC3626529

[6] Delcher AL, Harmon D, Kasif S, White O, Salzberg SL 1999 Improved microbial gene identification with Glimmer. Nucleic Acids Res 27:4636–4641. doi:10.1093/nar/27.23.4636. 10556321 PMC148753

[7] Salzberg SL, Delcher AL, Kasif S, White O 1998 Microbial gene identification using interpolated Markov models. Nucleic Acids Res 26:544–548. doi:10.1093/nar/26.2.544. 9421513 PMC147303

[8] Lowe TM, Eddy SR 1997 tRNAscan-SE: a program for improved detection of transfer RNA genes in genomic sequence. Nucleic Acids Res 25:955–964. doi:10.1093/nar/25.5.0955. 9023104 PMC146525

[9] Lagesen K, Hallin P, Rødland EA, Staerfeldt HH, Rognes T, Ussery DW 2007 RNAmmer: consistent and rapid annotation of ribosomal RNA genes. Nucleic Acids Res 35:3100–3108. doi:10.1093/nar/gkm160. 17452365 PMC1888812

[10] Tatusov RL, Galperin MY, Natale DA, Koonin EV 2000 The COG database: a tool for genome-scale analysis of protein functions and evolution. Nucleic Acids Res 28:33–36. doi:10.1093/nar/28.1.33. 10592175 PMC102395

[11] Franceschini A, Szklarczyk D, Frankild S, Kuhn M, Simonovic M, Roth A, Lin J, Minguez P, Bork P, von Mering C, Jensen LJ 2013 String v9.1: protein-protein interaction networks, with increased coverage and integration. Nucleic Acids Res 41:D808–D815. doi:10.1093/nar/gks1094. 23203871 PMC3531103

[12] Harris MA, Clark J, Ireland A, Lomax J, Ashburner M, Foulger R, Eilbeck K, Lewis S, Marshall B, Mungall C, Richter J, Rubin GM, Blake JA, Bult C, Dolan M, Drabkin H, Eppig JT, Hill DP, Ni L, Ringwald M, Balakrishnan R, Cherry JM, Christie KR, Costanzo MC, Dwight SS, Engel S, Fisk DG, Hirschman JE, Hong EL, Nash RS, Sethuraman A, Theesfeld CL, Botstein D, Dolinski K, Feierbach B, Berardini T, Mundodi S, Rhee SY, Apweiler R, Barrell D, Camon E, Dimmer E, Lee V, Chisholm R, Gaudet P, Kibbe W, Kishore R, Schwarz EM, Sternberg P, 2004 The Gene Ontology (GO) database and informatics resource. Nucleic Acids Res 32:D258–D261. doi:10.1093/nar/gkh036. 14681407 PMC308770

[13] Hamon MA, Lazazzera BA 2001 The sporulation transcription factor Spo0A is required for biofilm development in *Bacillus subtilis*. Mol Microbiol 42:1199–1209. doi:10.1046/j.1365-2958.2001.02709.x. 11886552

[14] Davey ME, Duncan MJ 2006 Enhanced biofilm formation and loss of capsule synthesis: deletion of a putative glycosyltransferase in *Porphyromonas gingivalis*. J Bacteriol 188:5510–5523. doi:10.1128/JB.01685-05. 16855241 PMC1540017

[15] Swain MR, Ray RC 2009 Biocontrol and other beneficial activities of *Bacillus subtilis* isolated from cowdung microflora. Microbiol Res 164:121–130. doi:10.1016/j.micres.2006.10.009. 17320363

[16] Lian LH, Tian BY, Xiong R, Zhu MZ, Xu J, Zhang KQ 2007 Proteases from *Bacillus*: a new insight into the mechanism of action for rhizobacterial suppression of nematode populations. Lett Appl Microbiol 45:262–269. doi:10.1111/j.1472-765X.2007.02184.x. 17718837

[17] Kim PI, Chung K-C 2004 Production of an antifungal protein for control of *Colletotrichum lagenarium* by *Bacillus amyloliquefaciens* MET0908. FEMS Microbiol Lett 234:177–183. 15109737 10.1016/j.femsle.2004.03.032

[18] Pozo MJ, Baek JM, García JM, Kenerley CM 2004 Functional analysis of *tvsp1*, a serine protease-encoding gene in the biocontrol agent *Trichoderma virens*. Fungal Genet Biol 41:336–348. doi:10.1016/j.fgb.2003.11.002. 14761794

[19] López-Maury L, Florencio FJ, Reyes JC 2003 Arsenic sensing and resistance system in the cyanobacterium *Synechocystis* sp. strain PCC 6803. J Bacteriol 185:5363–5371. doi:10.1128/JB.185.18.5363-5371.2003. 12949088 PMC193754

[20] Andersen SJ, Quan S, Gowan B, Dabbs ER 1997 Monooxygenase-like sequence of a *Rhodococcus equi* gene conferring increased resistance to rifampin by inactivating this antibiotic. Antimicrob Agents Chemother 41:218–221. 8980786 10.1128/aac.41.1.218 PMC163691

[21] Gioia J, Yerrapragada S, Qin X, Jiang H, Igboeli OC, Muzny D, Dugan-Rocha S, Ding Y, Hawes A, Liu W, Perez L, Kovar C, Dinh H, Lee S, Nazareth L, Blyth P, Holder M, Buhay C, Tirumalai MR, Liu Y, Dasgupta I, Bokhetache L, Fujita M, Karouia F, Eswara Moorthy P, Siefert J, Uzman A, Buzumbo P, Verma A, Zwiya H, McWilliams BD, Olowu A, Clinkenbeard KD, Newcombe D, Golebiewski L, Petrosino JF, Nicholson WL, Fox GE, Venkateswaran K, Highlander SK, Weinstock GM 2007 Paradoxical DNA repair and peroxide resistance gene conservation in *Bacillus pumilus* SAFR-032. PLoS One 2:e928. doi:10.1371/journal.pone.0000928. 17895969 PMC1976550

